# Supplementary material for: Compound Heterozygous Mutations of SACS in a Korean Cohort Study of Charcot-Marie-Tooth Disease Concurrent Cerebellar Ataxia and Spasticity
Source: Int J Mol Sci. 2024 Jun 9;25(12):6378. doi: 10.3390/ijms25126378 (PMC11204044; doi:10.3390/ijms25126378)
Supplement: Supplementary file 1 [file ijms-25-06378-s001.zip › ijms-3037742-supplementary.pdf]

Table S1. Evaluation of the *SACS* variants by the ACMG/AMP criteria.

Variants		Criteria	Evaluation
**Nucleotides [1]**	**Amino acids [1]**		
c.1966_1967insT	[p.S656Ffs*1]	PVS1 + PM2 + PM3 + PP1 + PP4	P
c.4138C>G	p.P1380A	PM1 + PM2 + PM3 + PP1 + PP4 + PP3 + BP1	P
c.2439_2440delAT	p.V815Gfs*2	PVS1 + PM2 + PM3 + PP1 + PP4	P
c.10897T>G	p.F3633V	PM1 + PM2 + PM3 + PP1 + PP4 + PP3 + BP1	LP
c.2903_2906delACAG	p.D968Vfs*13	PVS1 + PM2 + PM3 + PP1 + PP4	P
c.13217delC	p.T4406Rfs*45	PVS1 + PM2 + PM3 + PP1 + PP4	P
c.1596T>A	p.Y532X	PVS1 + PM2 + PM3 + PP1 + PP4	P
c.3159_3160delCT	p.F1054X	PVS1 + PM2 + PM3 + PP1 + PP4	P

Abbreviations: ACMG/AMP: American College of Medical Genetics and Genomics and Association for Molecular Pathology, LP: likely pathogenic, P: pathogenic. [1] GenBank accession numbers of nucleotide and amino acid reference sequences are NM_014363.6 and NP_055178.3.

Table S2. Electrophysiological features in the patients with *SACS* mutations.

**Patients**	**FC591 (III-1)**		**FC591 (III-2)**		**FC937 (II-1)**		**FC1157 (II-2)**		**FC1157 (II-4)**		**FC1176(II-1)**	
Exam. age (yrs)	35		33		27		26		26		22	
Site	Rt	Lt	Rt	Lt	Rt	Lt	Rt	Lt	Rt	Lt	Rt	Lt
Motor nerve conduction study												
Median nerve												
DTL (ms)	5.5	5.8	4.7	5.3	5.8	6.3	6.6	6.0	4.6	4.6	6.2	4.9
CMAP (mV)	5.7	5.6	3.3	7.4	12.6	5.2	7.5	9.2	12.2	14.4	14.1	13.6
MNCV (m/s)	39.2	38.1	43.2	47.8	40.0	36.0	39.0	33.0	42.0	40.6	42.0	46.0
Ulnar nerve												
DTL (ms)	3.3	3.3	3.1	3.4	3.5	3.2	3.7	4.0	3.4	3.3	3.3	3.3
CMAP (mV)	11.4	9.4	10.1	8.4	10.5	16.2	15.3	14.1	14.3	13.6	13.4	12.1
MNCV (m/s)	42.1	38.3	39.4	35.9	40.0	38.0	37.0	35.0	40.6	37.5	40.0	42.0
Peroneal nerve												
DTL (ms)	9.3	8.1	10.0	9.7	7.8	A	10.4	7.9	A	A	10.6	9.5
CMAP (mV)	2.9	2.0	0.2	0.3	1.8	A	0.7	1.6	A	A	0.9	1.6
MNCV (m/s)	24.1	23.4	20.0	15.8	23.0	A	22.0	29.0	A	A	26.0	24.0
Tibial nerve												
DTL (ms)	A	A	5.9	8.6	7.7	A	7.5	7.1	6.2	5.4	7.5	7.7
CMAP (mV)	A	A	0.3	0.4	0.6	A	3.5	4.7	3.5	4.9	5.3	5.2
MNCV (m/s)	A	A	24.8	19.2	27.0	A	28.0	30.0	31.7	30.1	31.0	34.0
Sensory nerve conduction study												
Median nerve												
SNAP (μV)	A	A	A	A	1.6	A	A	A	A	A	0.5	0.5
SNCV (m/s)	A	A	A	A	35.0	A	A	A	A	A	27.0	22.0
Ulnar nerve												
SNAP (μV)	A	A	A	A	0.8	1.7	A	A	A	A	A	1.0
SNCV (m/s)	A	A	A	A	37.0	32.0	A	A	A	A	A	34.0
Sural nerve												
SNAP (μV)	A	A	A	A	1.5	1.0	A	A	A	A	2.4	3.6
SNCV (m/s)	A	A	A	A	36.0	35.0	A	A	A	A	26.0	28.0

Abbreviations: A: absent potential, CMAP: compound muscle action potential, DTL: distal terminal latency, Lt: left side, MNCV: motor nerve conduction velocity, Rt: right side, SNAP: sensory nerve action potential, SNCV: sensory nerve conduction velocity. Normal values: motor DTLs are < 3.6 ms (median), < 2.5 ms (ulnar), < 4.8 ms (peroneal), and < 5.1 ms (tibial). Motor NCVs are ≥ 50.5 m/s (median), ≥ 51.1 m/s (ulnar), ≥ 41.2 m/s (peroneal), and ≥ 41.1 m/s (tibial). Sensory NCVs are ≥ 39.3 m/s (median), ≥ 37.5 m/s (ulnar), and ≥ 32.1 m/s (sural). Motor amplitudes are ≥ 6 mV (median), ≥ 8 mV (ulnar), ≥ 1.6 mV (peroneal), and ≥ 6 mV (tibial). Sensory amplitudes are ≥ 8.8 μV (median), ≥ 7.9 μV (ulnar), and ≥ 6.0 μV (sural).

Table S3. Thigh and calf MRI features of the patients with *SACS* mutations.

Patients	FC937 (II-1)		FC1157 (II-2)		FC1157 (II-4)		FC1176 (II-1)	
Sex/ages at exam (yrs)	Female / 27		Female / 26		Male / 25		Male / 21	
Level	Prox	Distal	Prox	Distal	Prox	Distal	Prox	Distal
Thigh muscles								
Anterior compartment								
Sartorius	2/2	2/2	1/1	1/1	0/0	1/1	2/2	2/2
Rectus femoris	1/1	1/1	0/0	0/0	0/0	0/0	0/0	0/0
Vastus intermedius	1/1	1/1	0/0	0/0	0/0	0/0	1/1	0/1
Vastus lateralis	2/1	2/2	0/0	0/0	0/0	0/0	1/1	1/1
Vastus medialis	1/1	1/1	0/0	0/0	0/0	0/0	0/0	1/1
Medial compartment								
Adductor longus	1/1	N/A	0/0	N/A	0/0	N/A	0/0	N/A
Adductor brevis	1/1	N/A	1/1	N/A	0/0	N/A	1/1	N/A
Adductor magnus	1/1	N/A	1/1	N/A	0/0	N/A	1/1	N/A
Gracilis	2/2	2/2	1/1	1/1	0/0	1/1	1/1	1/1
Posterior compartment								
Semitendinosus	2/2	2/2	1/1	1/1	1/1	1/1	2/1	1/1
Semimembranosus	2/2	2/2	1/1	1/1	0/0	1/1	1/1	2/2
Biceps femoris	2/2	2/2	1/1	1/1	0/0	1/1	1/1	2/2
Calf muscles								
Anterior compartment								
Tibialis anterior	2/1	1/1	0/0	0/0	0/0	0/0	1/1	0/0
Extensor digitorum longus	1/1	1/1	0/0	0/0	0/0	0/0	1/1	1/1
Extensor hallucis longus	N/A	1/1	N/A	0/0	N/A	0/0	N/A	1/1
Lateral compartment								
Peroneus longus	1/1	1/1	0/0	0/0	0/0	0/0	1/1	1/1
Superficial posterior compartment								
Gastrocnemius medial	1/1	N/A	0/0	N/A	0/0	N/A	1/1	N/A
Gastrocnemius lateral	1/1	N/A	0/0	N/A	0/0	N/A	1/1	N/A
Soleus medial	1/1	1/1	1/1	0/0	0/0	0/0	1/1	2/2
Soleus lateral	1/1	1/1	0/0	0/0	0/0	0/0	1/1	1/2
Deep posterior compartment								
Tibialis posterior	0/0	0/0	0/0	0/0	0/0	0/0	0/0	0/0
Flexor digitorum longus	N/A	1/1	N/A	0/0	N/A	0/0	N/A	0/0
Flexor hallucis longus	N/A	1/1	N/A	0/0	N/A	0/0	N/A	0/0

Abbreviation: Prox: proximal. The presence of fatty infiltration based on a five-point semiquantitative scale: grade 0: normal, grade 1: some fatty streaks, grade 2: less fat than muscle, grade 3: fatty degeneration of 50%, and grade 4: fatty in filtration of more than 50%.
